# Annotations

**Published:** 1907-06-15

**Authors:** 


### ANNOTATIONS.

### The Royal Society of Medicine.

The Royal Society of Medicine is now an accomplished fact, and arrangements are already in progress to secure active work by the various sections at the commencement of next session. There have been some disappointments, as is well known, but assent to the amalgamation has been given by 14 societies, so that the centripetal movement has evidently commanded a wide measure of approval. The latest Society to give in its adhesion is the Therapeutical Society, and this is a decided gain. Though this Society has only been in existence some five years, it has proved itself one of the most energetic and enterprising of our medical organisations. Its published transactions show the accomplishment of much good work, and it cultivates a department of medicine which is sometimes neglected in societies having a more specialised ambition. As an indication of its enterprise, note may be taken of the opportunities it offers to members to visit University and other laboratories where pharmacological work is specially cultivated. Last year the Society in this fashion met at Cambridge, and had the advantage of a series of demonstrations by Professor Dixon. This year, on July 6th, a visit is to be paid to Oxford, where Professor Gotch and Dr. Ainley Walker will show a series of pharmacological experiments in the University laboratories. With such a record the future of the therapeutical section can be counted on with confidence. Reverting to the new Royal Society of Medicine, it is satisfactory to note, from a statement issued by the honorary secretaries, that the financial position is secure. This is a part of the amalgamation scheme which has excited much criticism, and it is pleasing to know that anxiety on this point is no longer necessary. In these columns we have steadily supported the proposal for amalgamation, and now it is realised we heartily congratulate those who have worked hard to accomplish it.

### Ophthalmology at Oxford.

The annual course on ophthalmology at Oxford has become an established and permanent feature of the educational opportunities offered by the University Medical School. On the present occasion the course is to extend from July 15th to 27th. As in former years, the scheme proposes to include the whole range of ophthalmology, and to demonstrate the various aspects of the subject, so far as possible, on actual patients. It is anticipated that some 500 cases will be shown in the various clinics that have been arranged. A considerable part of the systematic instruction will be undertaken by the University Reader in Ophthalmology, and there will be daily practical work at the hospital, in which the staff of the hospital will take part. In this way will be met the wants of those to whom ophthalmological work is, in any practical sense, almost or entirely an unknown department. The latter half of the course will include a series of lectures by a number of well-known ophthalmic surgeons, and the clinical opportunities will be continued. Professor Wm. Osler will give several clinical lectures at the Radcliffe Infirmary, and the medical aspects of ophthmology are to receive further emphasis in a lecture and clinical demonstration by Dr. Risien Russell. Altogether the programme is a most attractive one, and it may be cordially commended to all who desire to cultivate a practical acquaintance with ophthalmology. The wants of more advanced students are not to be neglected, and the clinical material collected for purposes of study will appeal to many who have already acquired a fair body of experience. The fee for the course is five guineas, and anyone wishing to attend should communicate with the University Reader in Ophthalmology, Mr. R. W. Doyne, M.A., 34 Weymouth Street, W.

### Royal Visits and Guardians' Extravagance.

One of the most unpleasing of the revelations made about the extravagance connected with the Hammersmith Workhouse is the explanation given of an expenditure of £457 for distempering, when the estimate for the work was only £50. Mr. Herbert Gough, a member of the firm of architects that designed the building, explained that Princess Henry of Battenberg having graciously consented to come and open the building, one ward was done up as "a show ward," and, moreover, this "show ward" managed to "find its way over the entire building." In connection with the Royal visit there were also charges amounting in all to £836. The arrangements were left to the builder, as, Mr. Gough explained, is the custom. Mr. Oxley, the Local Government Board Inspector, gave a further explanation of a more illuminating character when he added that the builder got a commission of 10 per cent. on this expenditure. There is something shocking in the idea that a Royal visit, paid in the spirit of true charity and sympathy with the poor, should thus be made an occasion for exploiting the ratepayers. Our Royal Family, when they visit a charitable institution, do not want to see "show wards." The managers of many institutions could tell of visits paid without warning, or so nearly so as to render impossible special preparations being made to deceive the visitors, and they could tell also of requests—those requests which are equivalent to commands—to be taken to those parts of the building where strangers are rarely conducted, and whose importance is purely practical. To fix up one part of a building in a more decorative way than the rest in order to impress the Princess was a sufficient misapprehension of the meaning of her presence at the workhouse; but to make her visit an excuse for running up expenses in the fashion that was done at Hammersmith was a far worse offence. Certain courtesies are desirable when a member of the Royal Family honours an institution with a visit, but these are not of necessity costly, and though Guardians are not, as a rule, wealthy people, their united efforts might easily have sufficed to provide all that was required. To expend £836 of ratepayers' money was wrong and unnecessary. No one could have desired it except perhaps those who made money out of the business, and took a commission on the wasteful expenditure.

